# Hyperkinetic Movement Disorder as the First Manifestation of Moyamoya Disease in a 15‐Year‐Old: A Case Report

**DOI:** 10.1002/ccr3.71878

**Published:** 2026-04-27

**Authors:** Lina Okar, Reema Gowda, Hadeel Alzoubi, Karthik Narayanan, Maxwell Wallace

**Affiliations:** ^1^ Department of Neurology SSM Health/Saint Louis University Saint Louis Missouri USA; ^2^ Saint Louis University St. Louis Missouri USA; ^3^ Sidra Hospital and Research Center Doha Qatar

**Keywords:** chorea, hyperkinetic movement disorder, moyamoya, pediatrics movement disorders, vascular

## Abstract

Physicians evaluating pediatric movement disorders, especially chorea, should consider a broad differential diagnosis, including vascular etiologies such as moyamoya disease. Prompt recognition, appropriate neuroimaging, and early diagnosis are crucial for guiding management and optimizing patient outcomes.

## 1 Introduction

Moyamoya disease (MMD) is a rare, chronic cerebrovascular disorder characterized by progressive narrowing of the terminal portion of the internal carotid artery (ICA) and the proximal segments of the middle cerebral artery (MCA) and anterior cerebral artery (ACA). While predominantly affecting the anterior circulation, posterior circulation involvement has been described as well [1, 2]. Abnormal collateral vascularization develops as a compensatory mechanism, producing the characteristic “puff of smoke” appearance on angiography [3]. MMD exhibits a bimodal distribution, with incidence peaks in childhood (around age five) and adulthood (in the fourth decade), and shows a female predominance with a 2:1 ratio [2]. Although its etiology remains unclear, risk factors include a higher prevalence of Eastern Asian descent due to genetic predisposition mutations in the RNF213 gene, and underlying autoimmune processes [2, 3].

Clinically, ischemic events are more common than hemorrhagic events, especially in pediatrics [2]. Movement disorders, though rare, have been reported in MMD, with presentations such as chorea, hemiballismus, limb‐shaking, and dystonia [4, 5]. Chorea, the most frequently reported movement disorder, is predominantly seen in pediatric populations, with an estimated prevalence of 3%–6% [6, 7]. The pathophysiology underlying these disorders is not fully understood but may involve ischemic changes disrupting basal ganglia function, hypertrophied collateral vessel networks, and hypermetabolic activity in the basal ganglia‐thalamocortical circuits observed in 18F‐FDG PET imaging [8, 9, 10]. The definitive diagnosis of MMD relies on catheter angiography. While no treatment prevents disease progression, therapeutic strategies aim to mitigate secondary complications. Antiplatelet agents can be used to reduce thrombotic risk, and corticosteroids have been reported as a bridging therapy to restore perfusion and modulate neurotransmitter activity. Surgical revascularization remains the mainstay of treatment, targeting restoration of cerebral blood flow [6, 11].

## 2 Case History/Examination

A 15‐year‐old Caucasian girl presented with 4 months of abnormal movements in the left arm, leg, and face, primarily affecting her arm, which worsened over the last three weeks. Movements are sometimes painful and triggered by activity and stress, interfere with her daily life, and disappear during sleep. She reports fatigue and unintentional weight loss but has no fever, joint issues, or skin sensitivity. Past medical history includes migraines which spontaneously remitted with menarche at age 11 and syncopal episodes at age 12, characterized by a prodrome of vision loss and tingling, followed with loss of consciousness for less than 30 s, then 5 min of slurred speech following the event. Family history includes migraines and lupus in the mother. Examination revealed jerky/writhing movements resembling mixed chorea and dystonia (Video 1). Gait was impaired by intermittent left foot plantarflexion due to dystonic movements. Over the next two months, her symptoms worsened, and she began to struggle academically due to the impact of movements in her school‐related activities.

**VIDEO 1:** Choreoathetosis starts distally in the left wrist and spreads proximally. Dystonic posturing of the trunk. Additionally, dystonia can be seen in the right arm. Video content can be viewed at https://onlinelibrary.wiley.com/doi/10.1002/ccr3.71878.

## 3 Differential Diagnosis

The patient underwent an extensive workup. MRI brain showed some white matter T2 hyperintensities, but this was obscured by movement artifact. serum tests included anti‐deoxyribonuclease‐B antibody (DNASE B Ab negative) and Anti‐streptolysin titers (ASO), which was slightly elevated initially but normal on a repeated checks. A Serum studies, including CBC, CMP, ESR, ANA, Anticardiolipin Ab, Ceruloplasmin, Copper, Iron, Ferritin, RPR, HIV, peripheral smear for acanthocytes, amino acids, lactate, pyruvate, acylcarnitine, VLCFA, and ammonia, The only positive test was ANA titer 1:640. Further rheumatologic workup was negative for Anti‐ds‐DNA antibodies, Anti‐ SSA/SSB, Complement C3, C4, RNP Ab, Chromatin Ab, ANCA vasculitis panel, CRP, thyroid Ab panel, and Anti‐tissue transglutaminase were unremarkable. Urine tests including toxicology, HCG, and organic acids, were unremarkable. Electrocardiography and Echocardiography were also unremarkable. Huntington's gene panel was negative.

**FIGURE 1:**
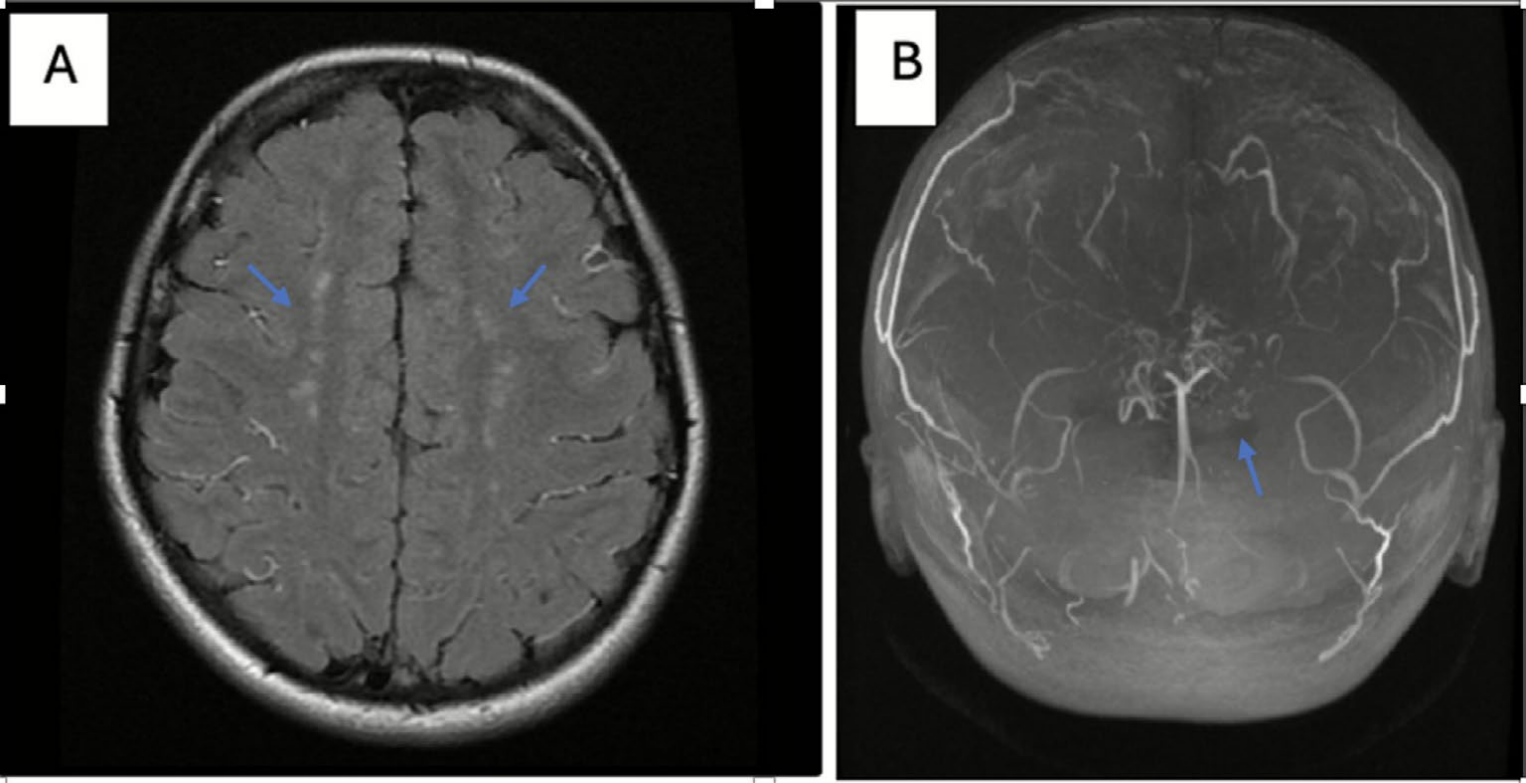
(A) MRI of the brain showed increased T2 and Flair signals foci in frontal subcortical and periventricular white matter. (B) MRA showing Narrowing of Anterior, Middle, and Posterior Cerebral Arteries.

In the setting of positive ANA and clinical worsening of the movements, developing chronic headaches and subjective cognitive changes, a decision was made to treat empirically with high‐dose corticosteroid (20 mg/kg/day for 3 days) due to concern of autoimmune CNS related disease. An MRI of the brain obtained Figure 1A with and without contrast showed increased T2 and FLAIR signal foci in the frontal subcortical and periventricular white matter.

## 4 Conclusion and Results: (Outcome and Follow‐Up)

**FIGURE 2:**
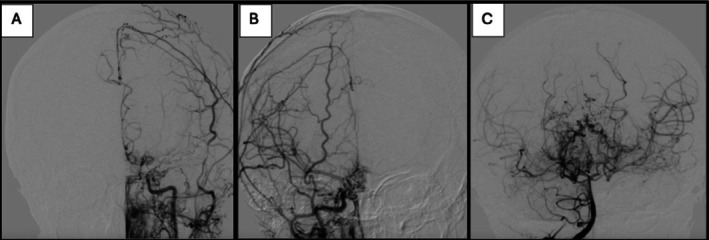
(A) Left common carotid angiogram, occlusion of the superclonoid segment, distal reconstitution at the carotid terminals from collateral filling from distal branches from the internal maxillary branch of the external carotid artery resulting in neovascularization. (B) Right common carotid angiogram, severe stenosis at the supraclinoid segment. The associated neovascularization is from collateral filling of enlarged lenticulostriate arteries. (C) Right vertebral artery angiogram. Posterior cerebral arteries are atretic at the P2 segment. There is neovascularization from enlargement of thalamoperforating and posterior choroidal arteries.

The MRA of the brain and neck revealed stenosis in the left internal carotid artery at the cavernous and supraclinoid segments, the origins of both internal carotid arteries. The narrowing extends to the anterior, middle, and posterior cerebral arteries, with evidence of collateral circulation Figure 1B. MRA with vessel wall protocol showed mild enhancement of only the proximal left internal carotid artery of uncertain clinical significance. The additional tests, including Digital Subtraction Angiography (DSA) Figure 2A–C, confirmed findings consistent with moyamoya arteriopathy. A CT angiography of the chest, abdomen, and pelvis showed no evidence of vasculopathy.

She had a near‐complete resolution of her movement disorder within days of starting the steroids, as well as improvement in headaches and cognitive symptoms. headaches began worsening within 1 month of weaning off steroids, so low dose maintenance steroids were started with good response. The overall constellation of findings, response to steroids, and severity of vascular abnormalities was concerning for possible CNS vasculitis at the time, so brain biopsy of the right frontal cortex 1 month after holding steroids did not show evidence of vasculitis. An Invitae hereditary moyamoya disease gene panel, Whole Exomes Sequences (WES) and Mito Panel results were also negative. CSF tests showed an increased protein of 57.

Surgical management included multi‐stage revascularization surgery including right‐sided myosynangiosis, left‐sided pial synangiosis, and bilateral occipital pial‐pericranial synangiosis. She experienced approximately 90% symptom improvement after surgery. Repeated MRI, MRA, and angiogram showed excellent reconstitution of the anterior circulation via the right myosynangiosis and left pial synangiosis. However, she developed a recurrence of her movement disorder, worsening headaches, fatigue, and cognitive slowing starting 2 months after her last stage of surgery, without any explanation on repeat imaging.

## 5 Discussion

Moyamoya incidence peaks at two age groups: children around 5 years of age and adults in their mid‐40s. Females are twice as affected as males, and familial occurrence is reported in about 15% of patients [12].

In this case, we highlight the atypical and rare presentation of Moyamoya disease in children. We described a 15‐year‐old girl who presented with a hyperkinetic movement disorder classified as dystonic‐choreiform. Even though she reported initial improvement to corticosteroid treatment, she eventually still required surgical management.

There is no medical therapy approved for MMD; however, multiple medications have been described, including aspirin, steroids, vasodilators, antibiotics, heparin, and calcium channel blockers, but none were validated. Secondary prevention is mainly surgical management [13]. It was proposed in previous case reports that steroids restore perfusion and modulate neurotransmitters within the basal ganglia. Steroids have been suggested as a potential treatment to alleviate symptoms while awaiting surgical intervention, which was seen in our case [6, 11]. Uncommon co‐occurrence of thyrotoxicosis and MMD was described in the literature with reported good response to setroid [14]. Although our case had normal thyroid function tests and negative thyroid antibodies screening, she responded to steroids. In alignment with previous literature, our case showed optimal, yet transient, response after surgical revascularization, achieving chorea improvements or resolution in a year post‐surgery [9]. Indirect method of revascularization, pial synangiosis, promotes the development of collateral circulation to the brain and decreases the risk of stroke, transient ischemic attacks, and seizures [15]. Follow‐up angiography demonstrates that pial synangiosis results in excellent postoperative collaterization of ischemic areas of the brain, which was evident in our case. After indirect revascularization, a follow‐up with DSA is done each year for bilateral MMD cases. In cases that are symptom‐free after surgical intervention, recent data suggest that MRI/MRA is just as effective as DSA for follow‐up care, while also reducing costs and potential DSA‐associated risks [14].

This case highlights the wide differentials physicians should keep in mind when approaching hyperkinetic movement disorders and considering MMD as a differential. Additionally, this case suggests that symptomatic response can be achieved temporarily with steroids while pursuing optimal surgical management. The role of steroids in movement disorder induced by MMD requires further studies to elucidate the mechanism behind the response and whether the response is related to comorbidity presence such as thyroid or autoimmune disease.

## Author Contributions

**Lina Okar:** conceptualization, writing – original draft, writing – review and editing. **Reema Gowda:** writing – original draft, writing – review and editing. **Hadeel Alzoubi:** writing – original draft, writing – review and editing. **Karthik Narayanan:** resources, writing – review and editing. **Maxwell Wallace:** resources, supervision, writing – original draft, writing – review and editing.

## Funding

The authors have nothing to report.

## Ethics Statement

Ethical approval is not required for this study in accordance with local or national guidelines. Written informed consent was obtained from the parent/legal guardian of the patient for publication of the details of their medical case and any accompanying images/video.

## Conflicts of Interest

The authors declare no conflicts of interest.

## Data Availability

All data generated or analyzed during this study are included in this article. Further inquiries can be directed to the first author (L.O.).
